# Evaluation of an integrated personalized feedback intervention for hazardous drinkers with elevated anxiety sensitivity and PTSD symptoms: Protocol for a randomized controlled trial

**DOI:** 10.1016/j.conctc.2023.101088

**Published:** 2023-02-04

**Authors:** Antoine Lebeaut, Eric R. Pedersen, David J. Francis, Michael J. Zvolensky, Anka A. Vujanovic

**Affiliations:** a Department of Psychology, University of Houston, Houston, TX, USA; b Department of Psychiatry and Behavioral Sciences, Keck School of Medicine, University of Southern California, Los Angeles, CA, USA

**Keywords:** PTSD, Alcohol, Anxiety sensitivity, Personalized feedback, Intervention, Efficacy

## Abstract

**Background:**

Hazardous drinking and posttraumatic stress disorder (PTSD) are commonly co-occurring conditions among adults. Motivational enhancement interventions, such as personalized feedback interventions (PFI), have demonstrated efficacy for reducing hazardous drinking. Emerging though scant literature has evaluated PFI for co-occurring PTSD and hazardous alcohol use. A transdiagnostic risk factor that may underlie this co-occurrence and inform novel PFI development is anxiety sensitivity (AS).

**Objective:**

To use a randomized controlled trial to evaluate the efficacy of a novel, computer-based PFI for hazardous drinkers with at least subclinical PTSD and elevated AS (AP-PFI), against a time-matched comparison condition (C-PFI).

**Methods:**

Participants (*N* = 100) will be recruited and enrolled from the Houston, TX community. The study includes: an in-person visit (baseline diagnostic assessment, a brief intervention, and a post-intervention assessment) and two follow-up assessments (1-week and 1-month). Participants who meet study inclusion criteria will be randomized to one of two conditions at baseline: AP-PFI or C-PFI. AP-PFI will consist of a brief, single-session, computer-delivered, PFI-based intervention that provides integrative and normative feedback about alcohol use, AS, and PTSD symptoms. C-PFI will be time-matched but will only include alcohol-related feedback.

**Conclusions:**

AP-PFI is designed to provide feedback about alcohol use, PTSD symptoms, and AS and their interplay and deliver psychoeducation on harm-reduction techniques, interoceptive exposure exercises, and stress management strategies. The intervention may address extant gaps in treatment for these co-occurring conditions by providing a brief, evidence-based, motivational enhancement intervention that is cost-effective with potential to be disseminated across a variety of healthcare settings.

## 1 Introduction

Hazardous drinking (i.e., a pattern of alcohol use that increases risk for adverse health consequences) [1] and posttraumatic stress disorder (PTSD) are highly prevalent and commonly co-occurring conditions [2] that are associated with greater disability and poor health outcomes as compared to either condition alone [3,4]. More than 25% of United States (U.S.) adults endorse hazardous drinking, which is one of the leading causes of preventable death in the U.S [5]. and globally [6]. Individuals who experience PTSD symptoms are approximately three times more likely to engage in hazardous drinking compared to those without PTSD symptomatology [7]. Integrative models of hazardous drinking-PTSD co-occurrence suggest bidirectional and transactional effects between PTSD symptoms and the maintenance and/or exacerbation of alcohol use over time [8]. Consensus regarding best practices for treating co-occurring hazardous drinking-PTSD is lacking [9,10], and low treatment-seeking rates have been found among hazardous drinkers with PTSD [2].

Generally, research on the behavioral treatment of PTSD and co-occurring substance use disorders is a highly understudied area [11]. Personalized feedback interventions (PFI) may help to address this gap in the literature. Drawing on motivational and social perspectives, PFI motivate behavior change via psychoeducation and the presentation of personalized (e.g., profiles of current health behaviors, assessment of risk severity) and normative feedback (i.e., comparisons to relevant sociodemographic groups) [12,13]. PFI target misperceptions regarding an individual's behaviors and actual normative behaviors, highlight consequences of these behaviors, and offer strategies for modifying them. PFI have demonstrated efficacy in reducing hazardous drinking and alcohol-related consequences across various populations [[14], [15], [16]] and offer a brief, cost-effective, easily disseminable [17] avenue for providing feedback and reducing alcohol use and consequences.

Given that PTSD symptoms may impede drinking reduction [18], and that hazardous drinkers with PTSD symptoms likely experience unique challenges to modifying their drinking [19] due to their increased reliance on alcohol to regulate negative emotional states, it is necessary to develop brief interventions that address hazardous drinking among trauma-exposed adults with PTSD symptoms. Accordingly, recent PFI work among military veterans with PTSD has evinced preliminary promising results on hazardous drinking and PTSD symptoms [16,[19], [20], [21]]. However, only three of these studies have explicitly integrated PTSD- and alcohol-focused personalized feedback and examined its impact on reducing hazardous drinking and PTSD symptoms [[19], [20], [21]]. Although these studies utilized either an app-based approach [19] or a single-session in-person format [20], no study to date has integrated these modalities into a single-session, comprehensive intervention for non-veteran, community adults. Moreover, it is important to consider malleable factors that underlie hazardous drinking-PTSD relations to enhance the efficacy of personalized evidence-based interventions.

One such transdiagnostic factor with established relevance to both hazardous drinking and PTSD is anxiety sensitivity (AS) [22]. AS, defined as the fear of anxiety-related sensations and cognitions (e.g., concerns related to increased heart rate and/or racing thoughts) [23,24], is a malleable [25], cognitive-affective vulnerability risk factor that is conceptually distinct from both trait anxiety [26] and PTSD [27]. AS has been positively related to hazardous drinking [[28], [29], [30]] and coping-oriented drinking motives across various populations [[31], [32], [33], [34]] and is related to increased rates of consumption and alcohol use disorder (AUD) [35,36]. Further, AS is implicated in the development and maintenance of PTSD [[37], [38], [39]] and may underlie hazardous drinking-PTSD comorbidity by amplifying PTSD symptomatology (e.g., hyperarousal) and motivating drinking to down-regulate such affect [22,40,41]. Despite the efficacy of AS interventions for reducing hazardous drinking [42] and PTSD symptoms [43], an integrated intervention to specifically target AS in the context of hazardous drinking and PTSD symptoms has not been developed or tested.

The objective of the present study is to examine the feasibility, acceptability, and efficacy of a novel PFI among hazardous drinkers with at least subclinical PTSD (i.e., endorsing at least two symptoms in each PTSD symptom cluster) and elevated AS. Hazardous drinkers with at least subclinical PTSD and elevated AS (*N* = 100) recruited from the community will be randomly assigned to receive Alcohol-PTSD-PFI (AP-PFI; *n* = 50) or active comparison condition (C-PFI; *n* = 50). The AP-PFI will provide feedback about alcohol use in the context of PTSD symptoms, PTSD-alcohol interplay, AS, and coping-oriented drinking. Primary outcomes include intervention-related perceived satisfaction and credibility/expectancies, drinking motivational factors (e.g., expectancies) and alcohol-related behaviors (e.g., cravings and consumption) and secondary outcomes include changes in AS and PTSD and exploring theoretically relevant mediators/moderators. Specific aims and hypotheses are described in Section 2.2.

## 2 Method

### 2.1 Trial design overview

The study is funded by the National Institute on Alcohol Abuse and Alcoholism (NIAAA; F31AA029600) and is registered on clinicaltrials.gov (ID: NCT04836442). Enrollment to the pilot randomized controlled trial (RCT) began in May 2021 and, as of this writing, is in the recruitment phase. This study will employ a longitudinal experimental design and include the following timepoints: (1) screening, (2) baseline assessment, (3) computer-delivered, one-session intervention (participants randomized to AP-PFI or C-PFI), (4) post-intervention follow-up assessment, (5) 1-week follow-up assessment, and (6) 1-month follow-up assessment.

### 2.2 Specific aims and hypotheses

The first aim of this project will be to evaluate the feasibility and acceptability of a brief, single-session, integrated, computer-based PFI (AP-PFI) based on participant ratings of satisfaction and the perceived credibility/expectancies of the intervention. The second aim will be to conduct a RCT to examine the efficacy and intervention effects on drinking, AS, and PTSD outcomes of AP-PFI, versus C-PFI, across the post-intervention and 1-week and 1-month follow-ups. The third aim will be to explore mechanisms of change and moderators.

Regarding the first aim (feasibility and acceptability), it is hypothesized that participants randomized to AP-PFI (vs. C-PFI) will evince greater satisfaction and perceived credibility/expectancies of the intervention. Regarding the second aim (efficacy and intervention effects), it is hypothesized that participants randomized to AP-PFI (vs. C-PFI) will evince (1) greater motivation/intention to reduce drinking, (2) lower levels of AS, (3) lower frequency and quantity of alcohol consumption, (4) reduced negative consequences of drinking and alcohol-related craving, and (5) lower PTSD symptom severity at 1-week and 1-month follow-up. Additionally, regarding the third aim, it is hypothesized that the effects of AP-PFI (vs. C-PFI) on post-intervention outcomes will be mediated by greater motivation/intention to reduce drinking and lower levels of AS. It is also hypothesized that the effects of AP-PFI (vs. C-PFI) on post-intervention, 1-week, and 1-month follow-up outcomes will be moderated by biological sex (i.e., larger magnitude of effect among female participants, relative to male participants) and family history of AUD (i.e., larger magnitude of effect among participants with a family history of AUD).

### 2.3 Participant recruitment and eligibility

The proposed study will include 100 hazardous alcohol users with PTSD symptoms and elevated AS recruited from the Houston, Texas community. Potential participants will be assessed for inclusion/exclusion criteria via an online screener and during the baseline appointment. Inclusion criteria will include the following: (1) ≥21 years of age (demographics questionnaire), (2) current hazardous drinking (Alcohol Use Disorders Identification Test cut-off scores of ≥8 for males and ≥7 for females [44,45]), (3) lifetime exposure to a *Diagnostic and Statistical Manual of Mental Disorders, Fifth Edition* (*DSM-5*) Criterion A traumatic event (Life Events Checklist for *DSM*-5) and endorsing ≥2 symptoms in each *DSM-5* PTSD symptom cluster (PTSD Checklist for DSM-5 [46]), (4) elevated AS score (≥17 on Anxiety Sensitivity Index-3 [47]), and (5) fluency in English (demographics questionnaire). Exclusion criteria will include the following: (1) concurrent alcohol or other substance use treatment (demographics questionnaire), (2) current/past bipolar or psychotic disorder (demographics questionnaire), (3) current imminent risk of suicide (i.e., past-month suicidal ideation with intent/plan; Beck Suicide Scale), (4) current pregnancy (demographics questionnaire), (5) inability to provide verbal or written consent, and (6) breath analysis estimating blood alcohol concentration (BAC) above 0 at baseline (Alco-Sensor FST Breath Alcohol Tester). Participants with non-zero BAC at baseline will be rescheduled for another baseline appointment.

### 2.4 Procedures

Participants will be recruited from the Houston community through multimedia platforms (university listservs, flyers, etc.). Interested participants will complete an online screener via Qualtrics, an online data collection platform. Participants who qualify will be contacted by trained study staff who will describe the study and schedule the baseline visit. Upon arrival to the lab, participants will provide informed consent and be assessed regarding trauma history, PTSD symptomatology, substance use, and the presence of current/past bipolar or psychotic disorder, as determined by the MINI-International Neuropsychiatric Interview. A urine drug screen will be implemented at baseline to identify current substance use (not used for exclusion).

**Fig. 1:**
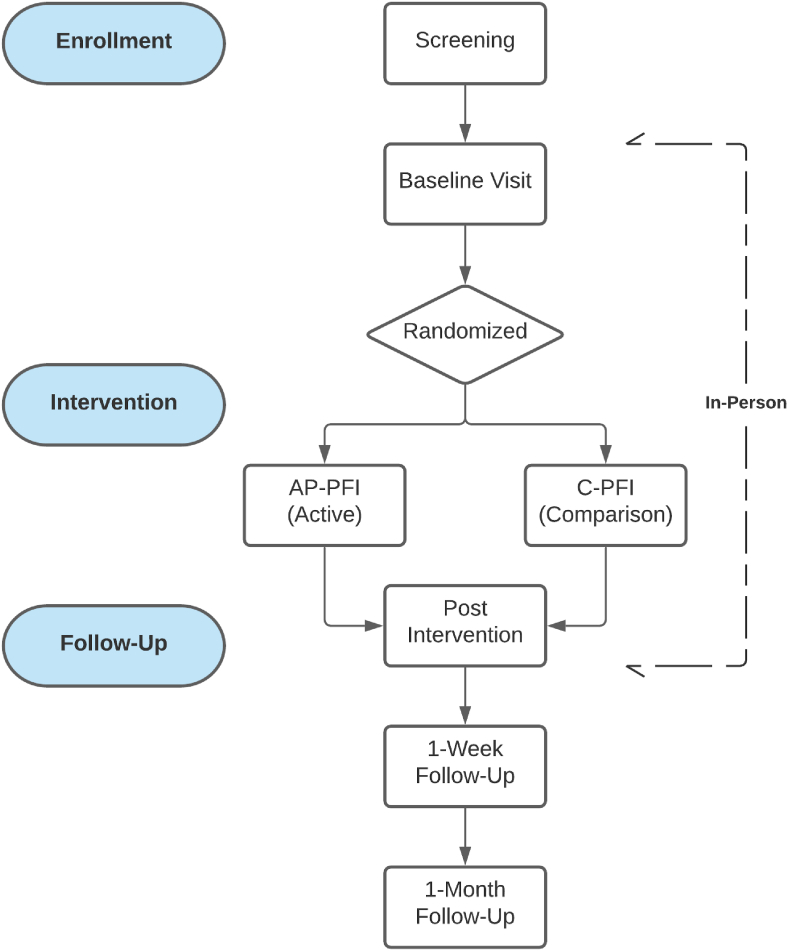
Study CONSORT flow diagram. Potential participants are screened to determine preliminary study eligibility. Eligible participants are invited for an in-person visit that includes (1) a baseline diagnostic and symptom assessment, (2) a brief intervention (randomized to one of two conditions: intervention or active comparison condition), and (3) a post-intervention assessment. Participants are then emailed follow-up surveys after 1-week and 1-month post-intervention.

Once eligibility is confirmed, participants will be randomly assigned (equally; 50 participants per condition, using a simple randomization design) to AP-PFI or C-PFI and complete the intervention/comparison condition on laboratory computers. Participants will then complete a post-intervention assessment. Online follow-up assessment will include similar surveys and will be scheduled 1-week and 1-month after completing the intervention conditions. Participants will be compensated $25 for completing the in-person baseline assessment and $35 and $40 for completing the 1-week and 1-month follow-up surveys, respectively, for a total of $100 in eGift cards. All study procedures were approved by the University Institutional Review Board and all study measures and assessment procedures are outlined in Table 1 and Fig. 1.

**Table 1:** Study measures and timepoints.

Measures	Items	Assessment Timepoint				
		**Screen**	**Baseline**	**Post**	**1-Week**	**1-Month**
Demographics Questionnaire	**20**	**X**				
Urinalysis drug screening	**N/A**		**X**			
Breath analysis	**N/A**		**X**			
MINI-International Neuropsychiatric Interview	**Variable**		**X**			
Treatment Credibility/Expectancy Questionnaire	**6**			**X**		
Modified Satisfaction with Treatment	**7**			**X**		
Alcohol Ladder	**1**		**X**	**X**	**X**	**X**
Modified Alcohol Use Disorders Identification Test	**3**–**10**	**X**	**X**		**X**	**X**
The Short Inventory of Problems	**15**		**X**		**X**	**X**
Alcohol Urges Questionnaire	**8**		**X**	**X**	**X**	**X**
Timeline Follow-Back	**30**		**X**		**X**	**X**
Life Events Checklist for *DSM-5*	**17**	**X**	**X**		**X**	**X**
Modified PTSD Checklist for *DSM-5*	**20**	**X**	**X**		**X**	**X**
Anxiety Sensitivity Index-3	**18**	**X**				
The Short Scale Anxiety Sensitivity Index	**5**		**X**		**X**	**X**
The Beck Suicide Scale	**21**	**X**	**X**		**X**	**X**

*Note.* MINI-International Neuropsychiatric Interview (MINI) [49]; Treatment Credibility/Expectancy Questionnaire (TCEQ) [58]; Modified Satisfaction with Treatment (SAT) [59]; Alcohol Ladder [53]; Alcohol Use Disorders Identification Test (AUDIT) [1], modified timeframe to correspond with each follow-up timepoint; The Short Inventory of Problems (SIP) [54]; Alcohol Urges Questionnaire – Short Form- Revised (AUQ) [55]; Timeline Follow-Back (TLFB) [52]; Life Events Checklist for DSM-5 (LEC-5) [50]; PTSD Checklist for DSM-5 (PCL-5) [46], modified timeframe to correspond with each follow-up timepoint; Anxiety Sensitivity Index-3 [56]; Short-Scale Anxiety Sensitivity Index (SSASI) [47]; Beck Suicide Scale (BSS) [57].

### 2.5 Intervention and comparison conditions

*Personalized feedback intervention condition (AP-PFI)*. Participants will receive a brief, 20-min computer-based AP-PFI. Both PFI will be time-matched and designed to facilitate engagement (e.g., dynamic/clickable content). Personalized content will be collected and dynamically imputed via Qualtrics and the total amount of time spent in either condition will be recorded. The content and process of the PFI will be grounded in established concepts such as the theory of planned behavior [48] and principles of motivational interviewing [13]. Specifically, AP-PFI will incorporate personalized profiles for PTSD (e.g., intensity, interference, goals for functioning); AS (e.g., beliefs about anxiety), and alcohol consumption and problems (e.g., drinking quantity/frequency, alcohol-related problems). PFI components will include normative feedback (e.g., age- and sex-specific drinking, contrasted with self-reported perceived norms); AS and PTSD feedback; and PTSD-alcohol feedback (e.g., feedback regarding positive expectations for alcohol anxiolysis). Sex- and age-specific normative data will be extracted from the extant literature. Strategies to manage and reduce drinking and AS and improve stress management are reviewed. See Table 2 for AP-PFI content.

**Table 2:** Integrated, single-session PFI (AP-PFI) content summary.

Introduction
• **→**Introduction to AP-PFI program and use of personalized information. • **→**Participants provide a goal related to their drinking and/or mental health (e.g., *“I want to reduce my drinking because it affects my marriage”; “I want to improve my mental health because I want to be more present with my family”; “I want to drink two fewer beers on the nights I drink”*).
**Section 1:Integrated Alcohol Feedback and Psychoeducation**
Personalized feedback on drinking patterns
• **→**Feedback based on self-reported (1) drinking pattern and (2) prediction of drinking patterns for the average person with PTSD symptoms. • **→**Corrective information is provided on how their prediction compares to actual drinking patters among adults with PTSD symptoms.
Personalized feedback on alcohol intoxication and metabolism
• **→**Feedback based on participant's estimated daily and weekly blood alcohol concentration (BAC) during and after drinking sessions based on self-reported drinking pattern. • **→**Feedback on their estimated metabolization of alcohol over time based on their height, weight, and biological sex.
Personalized feedback on alcohol-related expectances
• **→**Feedback based on self-reported positive and negative alcohol-related expectances (e.g., *“I am more accepted socially”; “I become clumsy or uncoordinated”*).
**Section 2:Integrated PTSD Feedback and Psychoeducation on Self-Medication**
Psychoeducation on alcohol use and PTSD
• **→**Information on trauma and PTSD symptoms and their associations with increased alcohol use and problematic drinking. • **→**Defining drinking-related coping and the role and consequences of self-medication.
Personalized feedback on PTSD symptoms
• **→**Feedback based on self-reported PTSD symptoms. • **→**Normative comparisons are provided based on community adults with PTSD symptoms who do not drink versus those who drink to highlight interactions between drinking and PTSD.
Personalized feedback on PTSD-related expectances for alcohol use
• **→**Feedback based on self-reported positive consequences of drinking on PTSD symptomatology (e.g., *“My bad dreams would decrease after a few drinks”; “After a few drinks, I would be less angry or on edge”*).
Psychoeducation on self-medication
• **→**Information on the self-medication cycle between trauma, PTSD symptoms, and drinking.
**Section 3:Integrated Anxiety Sensitivity Feedback and Psychoeducation**
Psychoeducation on anxiety sensitivity
• **→**Information on the physical, cognitive, and social dimensions of anxiety sensitivity.
Personalized feedback on anxiety sensitivity and psychoeducation
• **→**Feedback based on self-reported anxiety sensitivity symptoms. • **→**Normative comparisons are provided based on community adults who drink and report PTSD symptoms. • **→**Psychoeducation provided on associations between anxiety, PTSD, and drinking.
**Section 4: Strategies to Reduce Drinking and Improve Mental Health**
Strategies to manage and reduce drinking
• **→**Review and positive reinforcement of self-reported protective behavioral strategies already in use. • **→**Information on additional strategies that can be used to manage and reduce drinking (e.g., “*alternate alcoholic and nonalcoholic drinks”; “avoid drinking shots of liquor”*). • **→**Interactive ranking of which strategies the participant would be willing to try and likelihood of using it.
Strategies to manage stress and improve coping
• **→**Introduction and review of two breathing exercises focused on reducing and managing stress. • **→***In vivo* practice of these breathing exercises via online video instruction.
Strategies to reduce anxiety sensitivity
• **→**Introduction and review of interoceptive exposure exercises developed to decreased anxiety sensitivity. • **→**Participants are provided with detailed instructions for the following exercises: (1) narrow straw breathing, (2) over-breathing, and (3) head between legs/head-rush exercise. • **→**Interactive ranking of which exercises the participant would be willing to try and likelihood of using it.
**Section 5: Review**
• **→**Intervention content is briefly summarized and reviewed. • **→**A handout that includes all intervention content is provided to the participant at the end of the study visit.

*Comparison condition (C-PFI).* Participants in the time-matched comparison condition will receive personalized feedback on alcohol use but will not receive PTSD or AS-related personalized feedback. C-PFI will include alcohol-focused components identical to those provided in AP-PFI (e.g., alcohol profiles, normative feedback, strategies to manage and reduce drinking). Therefore, it will be possible to isolate the impact of personalized PTSD and AS feedback versus personalized alcohol feedback.

### 2.6 Study measures

#### 2.6.1 Demographics

*Demographic questionnaire*. Participants will be asked to self-report demographic information including sociodemographic factors, health and medical information, educational levels, and family history. The questionnaire will be used to describe the current sample and will be administered at the screening assessment timepoint.

#### 2.6.2 Diagnostic measure

*MINI-International Neuropsychiatric Interview* (MINI) [49]. The MINI is a brief, structured, clinician-administered diagnostic interview to thoroughly assess common *DSM-5* disorders using *DSM-5* diagnostic guidelines. Trained doctoral students will conduct diagnostic assessments and will be supervised regarding the accuracy of their diagnosis by the principal investigator and doctoral-level raters. The MINI will be administered at baseline and will be used to assess if any exclusionary criteria are met and will serve to document psychopathology within the sample.

#### 2.6.3 PTSD-related measures

*Life Events Checklist for DSM-5* (LEC-5) [50]. The LEC-5 is a 17-item, self-report questionnaire that is used to screen for 16 potentially traumatic events (e.g., natural disaster, combat, sexual assault, transportation accident) experienced at any time throughout the lifespan, including an additional item for “other” potentially traumatic events not listed. Respondents will be asked to indicate (via check mark) whether each listed event “happened to me,” “witnessed it,” “learned about it,” “part of my job,” or “not sure.” If participants endorse “happened to me,” “witnessed it,” or “part of my job” for a given item, this will be coded as positive exposure to the particular traumatic event type. Total number of exposure event types will be summed to produce a variable indicating overall trauma load. The LEC-5 will be administered during the screening assessment and will be modified for all follow-up timepoints to assess any recent trauma exposure since the previously completed timepoint.

*PTSD Checklist for DSM-5* (PCL-5) [46]. Respondents will be asked to complete the PCL-5 regarding the “worst” traumatic event endorsed on the LEC-5. The PCL-5 is a 20-item, self-report questionnaire that measures PTSD symptom severity. Each items reflects a symptom of PTSD as defined in the *DSM-5* [51]. Both past-month and past-week versions will be utilized to capture symptom change at baseline and follow-up timepoints, respectively. Respondents will be asked to rate each item on a 5-point Likert scale (0 = *Not at all* to 4 = *Extremely*) in regard to the frequency in which they have been bothered by the symptom in the past week or past month. Total summed symptom severity scores range from 0 to 80, with higher scores indicating higher symptom severity. Eligible participants will be required to endorse ≥2 symptoms (with ratings of 2 [“moderately”] or higher) in each *DSM-5* PTSD symptom cluster, which aligns with current guidelines [46]. The PCL-5 will be administered at all study timepoints.

#### 2.6.4 Alcohol-related measures

*Alcohol Use Disorders Identification Test* (AUDIT) [1]. The AUDIT is a 10-item, self-report, Likert-style screening instrument developed to assess alcohol-related problems and drinking patterns. Items on the AUDIT are summed to create a total score, with higher scores indicating increased alcohol use severity. Participants will be screened with the full AUDIT to assess past-year alcohol use and the first three items of the AUDIT [i.e., AUDIT-C; 45] will be administered at all other timepoints (except the post-intervention assessment) to evaluate hazardous drinking over time. Scores will be used to assess changes in drinking and examine changes from hazardous to non-hazardous drinking. The timeframe will be modified for each timepoint to assess hazardous drinking since the previous timepoint.

*Timeline Follow-Back* (TLFB) [52]. The TLFB is a calendar-based, self-report questionnaire that collects information on the pattern, variability, and magnitude of drinking among individuals over a specific timeframe. For the current study, participants will be asked to estimate their daily consumption within the past two weeks (anchored two weeks prior to the date of their baseline assessment). The timeframe will be modified for the 1-week and 1-month follow-up timepoints to assess daily drinking between timepoints.

*Alcohol Ladder* [53]. The Alcohol Ladder is a one-item, self-report, and reliable measure that assesses an individual's motivation to change their alcohol use. It contains 10 statements, listed in ranked order, that correspond to the following stages of change: pre-contemplation, contemplation, preparation, action, and maintenance. Higher scores on the Alcohol Ladder indicate greater motivation to change one's alcohol use. Participants will be instructed to select the statement that best corresponds to their current stage. The Alcohol Ladder will be administered at baseline and at each follow-up timepoint.

*The Short Inventory of Problems* (SIP) [54] is a 6-item, self-report measure of potential consequences of drinking that is derived from the 45-item Drinker Inventory of Consequences [54]. Respondents will be asked to rate each item on a 4-point Likert scale (0 = *Never* to 3 = *Daily or almost daily*) in regard how much alcohol has led to consequences across five domains: physical, social responsibility, intrapersonal, impulse control, and interpersonal. Items are summed to produce a total score, with higher scores on the SIP indicating greater alcohol-related consequences. The SIP will be administered at baseline and 1-week and 1-month follow-up assessment timepoints (modified timeframes).

*Alcohol Urges Questionnaire–Short Form-Revised* (AUQ) [55]. The AUQ is an 8-item, self-report questionnaire that measures cravings to consume alcohol. Items on the AUQ are scored using a 7-point Likert scale (1 = *Strong disagree* to 7 = *Strong agree*) regarding current alcohol-related cravings (e.g., “All I want to do now is have a drink”). Total scores are averaged, and higher scores represent greater severity of cravings. The AUQ will be used to examine participants’ levels of alcohol-related cravings at baseline and each follow-up timepoint.

#### 2.6.5 Affective measures

*Short-Scale Anxiety Sensitivity Index* (SSASI) [47]. The SSAI is a 5-item self-report measure that assesses anxiety-related interoceptive sensations and cognitions. The SSASI was derived from the original 18-item *Anxiety Sensitivity Index-3 (ASI-3)* [56]. Each item is rated using a 4-point Likert scale (0 = *Very little* to 4 = *Very much*) in regard to how much a respondent agrees with the specific statement being rated, with higher scores indicating greater severity of AS. For the current study, the total score of the SSASI (i.e., the sum of all 5 items) will be used to indicate the severity of an individual's AS and will be administered at each timepoint (except the post-intervention assessment) and the total score of the ASI-3 will be used to assess study inclusion criteria (elevated AS score [≥17]) during the screening timepoint.

*Beck Suicide Scale* (BSS) [57]. The BSS is a 21-tem, self-report measure that assesses the severity of an individual's suicidal ideation or desire to die. Respondent's rate, on a 3-point scale, the most accurate statement for the intensity of past-week suicidal ideation (e.g., “I have no wish to live”), with higher summed total scores indicating more severe suicide ideation. The BSS will be administered at each timepoint (except the post-intervention assessment) to assess suicide risk**.**

#### 2.6.6 Intervention-related measures

*Treatment Credibility/Expectancy Questionnaire* (TCEQ) [58]. The TCEQ is a 6-item, self-report, and reliable index [58] that will be used to assess perceptions of intervention credibility/expectancies, including satisfaction and acceptability of AP-PFI relative to C-PFI. The TCEQ will also be employed to assess how participants think the intervention will be successful in terms of reducing alcohol use, PTSD symptoms, and AS, while also assessing anticipated reductions in drinking, PTSD symptoms, and AS (0–100%). Items on the TCEQ are rated on Likert-style scales from 1 (*not at all*) to 9 (*very*), with higher summed scores indicating greater positive perceptions of the intervention received by the participant. The TCEQ will be administered in the post-intervention assessment.

*Satisfaction with Treatment* (SAT) [59]. The SAT is a 7-item, self-report measure that will be used to measure participants’ positive and negative experiences with the computerized format of the intervention. A modified version of the SAT will be used for the current study. Participants will be asked to report the degree of satisfaction with the PFI interventions (e.g., AP-PFI or C-PFI) using a Likert-style rating scale and qualitative-based questions. Likert-style rated items will be summed to produce a total satisfaction score, with higher scores indicating greater satisfaction with the intervention received by the participant. The SAT will be administered in the post-intervention assessment.

### 2.7 Data analytic plan

Data will first be examined for multivariate normality; the maximum likelihood (ML) estimator will be used if data are approximately normal, and robust maximum likelihood will be used if the data are not multivariate normal. Categorical outcomes (i.e., hazardous drinking status) will be estimated using the mean and variance adjusted weighted least squares estimator when appropriate. Missing data will be handled using direct ML techniques within MPlus [60] under a missing at random assumption [61]. Differences in key baseline characteristics between randomly assigned intervention condition groups will be assessed and significant characteristics will be used as covariates to determine the influence of any potential randomization failures on results. Latent growth models (LGM) will be used to model linear trajectories of change in outcomes. Evaluation of LGM model fit will be examined using fit diagnostics (i.e., standardized residuals) and fit statistics (i.e., root mean square error of approximation) following recommended cutoffs [62].

*Aim 1.* To evaluate the feasibility and acceptability of AP-PFI vs. C-PFI, initial metrics of feasibility and efficacy will be examined, including (1) recruitment/retention rates throughout the duration of the study, (2) intervention acceptability at post-intervention, intervention utilization at 1-week and 1-month follow-up, and (3) initial efficacy at post-intervention, 1-week, and 1-month follow-up. Retention between conditions will be assessed utilizing binary logistic regression to examine condition (0 = control; 1 = PFI) as a predictor of each follow-up completion (0 = missed; 1 = completed). Effect sizes will be calculated with Cohen's *d* for frequency and proportions [i.e., of completed follow-ups; 63]. Intervention acceptability will then be evaluated with condition as a predictor of acceptability items (i.e., TCEQ, SAT); theoretically relevant variables such age, sex, and racial/ethnic minority status will be included as covariates. Cohen's *d* for mean differences will be calculated [63].

*Aim 2*. Hypotheses for Aim 2 (i.e., efficacy and intervention effects) will be examined using regression-based approaches, effect sizes (with 95% confidence intervals [CI]), and LGM using Mplus [60,62,64]. We will first estimate between-group differences with regression-based approaches by calculating odds ratios or Cohen's *d* effect sizes (with 95% CI) for the primary post-intervention outcomes (i.e., motivation/intention to reduce drinking) and primary 1-week and 1-month follow-up outcomes (i.e., change in rates from hazardous to non-hazardous drinking, frequency and quantity of alcohol consumption and negative consequences of drinking, and PTSD symptomatology). We will then conduct a series of conditional LGMs to examine the impact of intervention condition over time on the 1-month follow-up outcomes. A dummy code representing intervention condition will be included in the model as a predictor of the slope factor to quantify the effect of the PFI on the outcomes.

*Exploratory aim 3*. Similar modeling procedures will be used to test mechanisms of change. After conducting univariate LGM to explore changes in the putative mediators (i.e., motivation/intention to reduce drinking and levels of AS) as a function of condition, we will conduct a series of parallel process LGMs to examine how changes in the mechanisms relate to changes in primary follow-up outcomes at each assessment point. The effects of changes in the mechanisms of change on the outcomes will be examined by specifying the slope factor for each potential variable as a predictor of the slope factor for each outcome. The indirect effects of condition on outcomes via the hypothesized mechanisms will be evaluated by bootstrapped CI. Further, given the purported interaction effect of sex and a family history of AUD, dummy coded variables (0 = female, 1 = male; 0 = no history, 1 = history; respectively) will be included as additional predictors of slope in the LGM parallel process models.

*Sample size and power.* Power calculations were performed to statistically determine the target sample size of *N* = 100 for this pilot study**.** Small-to-moderate effects of the AP-PFI on primary follow-up outcomes and the hypothesized mechanisms are expected, and moderate-to-large associations (*r* > .30) between changes in the mechanisms and changes in 1-month follow-up outcomes are expected. Monte Carlo simulation studies [65] with 10,000 repetitions to identify the sample sizes necessary to detect effects indicated that the targeted sample size of 100 participants would yield 80% power to detect small-to-moderate effects for primary study aims at alpha = .05. Additionally, extant guidelines for pilot RCT designs [66] indicate that a targeted sample size of 100 participants would yield 90% power to detect small-to-moderate effects for study aims set at alpha = .05. Thus, the target sample size is well above current guidelines for pilot studies in clinical research [67].

## 3 Discussion

This pilot RCT will examine the feasibility, acceptability, and efficacy of a novel computer-based PFI among a sample of community adults with hazardous drinking, at least subclinical PTSD, and elevated AS. The present study provides an innovative extension of previous research and has the potential to directly inform care for this underserved population. There is a prominent need for brief interventions to reduce hazardous drinking [68]. Indeed, brief interventions, which commonly use client-centered motivational approaches, have evinced utility and cost-effectiveness in reducing hazardous drinking across a variety of settings and provide opportunities for individual to reflect on changing their alcohol use behavior [68].

Although PFI has demonstrated efficacy in multiple settings to reduce alcohol-related problems and use [69,70], PTSD symptoms can directly affect efforts to reduce drinking [18], and thus reduce the efficacy and effectiveness of alcohol-focused interventions. While some recent work has begun integrating PTSD-related feedback into interventions for military veterans, none have been tested among community adults and/or directly target transdiagnostic processes (e.g., AS) despite their potential utility [42,71]. Thus, the present study will be the first to directly target hazardous drinking and AS among individuals with PTSD symptoms and provide integrated feedback into a traditional alcohol-PFI-framework. Further, because the intervention is computer-based, if efficacious, this study has the potential to be disseminated in healthcare settings and thereby improve access to evidenced-based care for hazardous drinking. Globally, there is an emphasis on developing new integrated behavioral interventions due to the scale of the hazardous drinking problem [5,72]. Developing a motivational enhancement approach that does not require specialist health care is, therefore, highly innovative, particularly when dealing with potential comorbidities and other barriers to treatment, including PTSD [73].

It is important to note several study limitations. First, given the in-person, laboratory setting of PFI delivery, ecological validity may be reduced. However, past work suggests that in-person PFI can be more effective [74] and thus, this approach may out-weight the costs that result from remote PFI delivery, including reduced attention and disruptive/distracting settings (e.g., loud workplaces, the presence of others, etc.). This approach also may mirror administration of PFI in primary care or medical settings. Second, due to the in-person component of the study as well as the target population (i.e., individuals with PTSD symptomatology, hazardous drinking patterns, and elevated AS), self-selection bias may influence study outcomes as individuals with greater psychological impairment may rule themselves out of participating [2]. Additionally, follow-up assessments utilize self-report (versus clinician-administered) questionnaires to facilitate participant retention and as a result, various biases (e.g., response bias, social desirability bias) may influence study outcomes. Third, the computerized intervention delivery will not be recorded or monitored considering the preliminary nature of the trial and therefore, future trials should incorporate more rigorous fidelity monitoring procedures to ensure participant attention and focus during the intervention.

Overall, **the** proposed pilot RCT will test the feasibility, acceptability, and efficacy of an integrated intervention that specifically targets AS in the context of hazardous drinking and PTSD symptoms. The proposed intervention builds upon and extends previous integrated PFI work [75] and allows for the direct comparison of an integrated PTSD/AS/hazardous drinking PFI to a traditional hazardous drinking only PFI. Pending primary and secondary outcomes, the present study may help address pertinent gaps in PTSD-AUD intervention research and provide a brief, cost-effective, and easily disseminable motivational enhancement approach for this underserved and vulnerable population.

## Declaration of competing interest

The authors declare that they have no known competing financial interests or personal relationships that could have appeared to influence the work reported in this paper.

## Data Availability

The dataset that will be generated and/or analyzed from the current protocol will not be publicly available but will be available from the corresponding author upon request.

## References

[1] Saunders J.B. (1993). Development of the alcohol use disorders identification test (AUDIT): WHO collaborative project on early detection of persons with harmful alcohol consumption-II. Addiction.

[2] Blanco C. (2013). Comorbidity of posttraumatic stress disorder with alcohol dependence among US adults: results from national epidemiological survey on alcohol and related conditions. Drug Alcohol Depend..

[3] Berenz E.C., Coffey S.F. (2012). Treatment of co-occurring posttraumatic stress disorder and substance use disorders. Curr. Psychiatr. Rep..

[4] Simpson T.L. (2019). Clinical presentations, social functioning, and treatment receipt among individuals with comorbid life-time PTSD and alcohol use disorders versus drug use disorders: findings from NESARC-III. Addiction.

[5] NIAAA (2018).

[6] WHO, *World Health* (2018).

[7] Adams R.E., Boscarino J.A., Galea S. (2006). Alcohol use, mental health status and psychological well-being 2 years after the World Trade Center attacks in New York City. Am. J. Drug Alcohol Abuse.

[8] Hawn S.E., Cusack S.E., Amstadter A.B. (2020). A systematic review of the self-medication hypothesis in the context of posttraumatic stress disorder and comorbid problematic alcohol use. J. Trauma Stress.

[9] Roberts N.P. (2015). Psychological interventions for post-traumatic stress disorder and comorbid substance use disorder: a systematic review and meta-analysis. Clin. Psychol. Rev..

[10] Roberts N.P. (2016). Psychological therapies for post-traumatic stress disorder and substance use disorder. Cochrane Database Syst. Rev..

[11] Vujanovic A.A., Back S.E. (2019).

[12] Bandura A., DiClemente R.J., Peterson J.L. (1994). Preventing AIDS: Theories and Methods of Behavioral Interventions.

[13] Miller W.R., Rollnick S. (2002). Motivational Interviewing: Preparing People for Change.

[14] Bewick B.M. (2010). Providing web-based feedback and social norms information to reduce student alcohol intake: a multisite investigation. J. Med. Internet Res..

[15] Neighbors C., Larimer M.E., Lewis M.A. (2004). Targeting misperceptions of descriptive drinking norms: efficacy of a computer-delivered personalized normative feedback intervention. J. Consult. Clin. Psychol..

[16] Pedersen E.R. (2017). A randomized controlled trial of a web-based, personalized normative feedback alcohol intervention for young-adult veterans. J. Consult. Clin. Psychol..

[17] Riper H. (2009). Curbing problem drinking with personalized-feedback interventions: a meta-analysis. Am. J. Prev. Med..

[18] Jordan H.R. (2019). Posttraumatic stress disorder symptoms and problematic alcohol use in college students: the moderating role of alcohol protective behavioral strategies and gender. Psychol Trauma.

[19] Livingston N.A. (2020). Changes in alcohol use, PTSD hyperarousal symptoms, and intervention dropout following veterans' use of VetChange. Addict. Behav..

[20] Luciano M.T. (2019). Posttraumatic stress disorder symptoms improve after an integrated brief alcohol intervention for OEF/OIF/OND veterans. Psychol Trauma.

[21] Luciano M.T. (2022). Open trial of a personalized feedback intervention and substance-free activity supplement for veterans with PTSD and hazardous drinking. J Behav Cogn Ther.

[22] Vujanovic A.A. (2018). Anxiety sensitivity in the association between posttraumatic stress and substance use disorders: a systematic review. Clin. Psychol. Rev..

[23] Reiss S. (1991). Expectancy model of fear, anxiety, and panic. Clin. Psychol. Rev..

[24] Reiss S. (1986). Anxiety sensitivity, anxiety frequency and the predictions of fearfulness. Behav. Res. Ther..

[25] Hovenkamp-Hermelink J.H.M. (2019). Anxiety sensitivity, its stability and longitudinal association with severity of anxiety symptoms. Sci. Rep..

[26] Taylor S., Koch W.J., Crockett D.J. (1991). Anxiety sensitivity, trait anxiety, and the anxiety disorders. J. Anxiety Disord..

[27] Marshall G.N., Miles J.N., Stewart S.H. (2010). Anxiety sensitivity and PTSD symptom severity are reciprocally related: evidence from a longitudinal study of physical trauma survivors. J. Abnorm. Psychol..

[28] Guillot C.R. (2018). Anxiety sensitivity components in relation to alcohol and cannabis use, motives, and problems in treatment-seeking cigarette smokers. Addict. Behav..

[29] Paulus D.J. (2017). The role of anxiety sensitivity in the relation between anxious arousal and cannabis and alcohol use problems among low-income inner city racial/ethnic minorities. J. Anxiety Disord..

[30] DeMartini K.S., Carey K.B. (2011). The role of anxiety sensitivity and drinking motives in predicting alcohol use: a critical review. Clin. Psychol. Rev..

[31] Novak A. (2003). Anxiety sensitivity, self-reported motives for alcohol and nicotine use, and level of consumption. J. Anxiety Disord..

[32] Stewart S.H., Zeitlin S.B. (1995). Anxiety sensitivity and alcohol use motives. J. Anxiety Disord..

[33] Stewart S.H., Zvolensky M.J., Eifert G.H. (2002). The relations of anxiety sensitivity, experiential avoidance, and alexithymic coping to young adults' motivations for drinking. Behav. Modif..

[34] Stewart S.H., Zvolensky M.J., Eifert G.H. (2001). Negative-reinforcement drinking motives mediate the relation between anxiety sensitivity and increased drinking behavior. Pers. Indiv. Differ..

[35] Schmidt N.B., Buckner J.D., Keough M.E. (2007). Anxiety sensitivity as a prospective predictor of alcohol use disorders. Behav. Modif..

[36] Stewart S.H., Peterson J.B., Pihl R.O. (1995). Anxiety sensitivity and self-reported alcohol consumption rates in university women. J. Anxiety Disord..

[37] Asnaani A. (2015). The relationship between anxiety sensitivity and posttraumatic stress disorder: what is the impact of nicotine withdrawal?. Cognit. Ther. Res..

[38] Naragon-Gainey K. (2010). Meta-analysis of the relations of anxiety sensitivity to the depressive and anxiety disorders. Psychol. Bull..

[39] Gutner C.A. (2013). Longitudinal course of anxiety sensitivity and PTSD symptoms in cognitive-behavioral therapies for PTSD. J. Anxiety Disord..

[40] Gillihan S.J., Farris S.G., Foa E.B. (2011). The effect of anxiety sensitivity on alcohol consumption among individuals with comorbid alcohol dependence and posttraumatic stress disorder. Psychol. Addict. Behav..

[41] Lebeaut A., Tran J., Vujanovic A.A. (2020). Posttraumatic stress, alcohol use severity, and alcohol use motives among firefighters: the role of anxiety sensitivity. Addict. Behav..

[42] Watt M. (2006). Brief CBT for high anxiety sensitivity decreases drinking problems, relief alcohol outcome expectancies, and conformity drinking motives: evidence from a randomized controlled trial. J. Ment. Health.

[43] Allan N.P. (2015). Direct and mediating effects of an anxiety sensitivity intervention on posttraumatic stress disorder symptoms in trauma-exposed individuals. Cognit. Behav. Ther..

[44] Babor T.F. (1992).

[45] Bush K. (1998). The AUDIT alcohol consumption questions (AUDIT-C): an effective brief screening test for problem drinking. Arch. Intern. Med..

[46] Blevins C.A. (2015). The posttraumatic stress disorder checklist for DSM-5 (PCL-5): development and initial psychometric evaluation. J. Trauma Stress.

[47] Zvolensky M.J. (2018). Refinement of anxiety sensitivity measurement: the Short scale anxiety sensitivity index (SSASI). Psychiatr. Res..

[48] Ajzen I. (1991). The theory of planned behavior. Organ. Behav. Hum. Decis. Process..

[49] Sheehan D.V. (1998). The Mini-International Neuropsychiatric Interview (M.I.N.I.): the development and validation of a structured diagnostic psychiatric interview for DSM-IV and ICD-10. J. Clin. Psychiatr..

[50] Weathers F.W. (2013). http://www.ptsd.va.gov.

[51] American Psychiatric Association (2013).

[52] Sobell L.C., Sobell M.B. (1992). Measuring Alcohol Consumption: Psychosocial and Biochemical Methods.

[53] Hogue A., Dauber S., Morgenstern J. (2010). Validation of a contemplation ladder in an adult substance use disorder sample. Psychol. Addict. Behav..

[54] Miller W.R., Tonigan J.S., Longabaugh R. (1995).

[55] Bohn M.J., Krahn D.D., Staehler B.A. (1995). Development and initial validation of a measure of drinking urges in abstinent alcoholics. Alcohol Clin. Exp. Res..

[56] Taylor S. (2007). Robust dimensions of anxiety sensitivity: development and initial validation of the Anxiety Sensitivity Index-3. Psychol. Assess..

[57] Beck A.T., Steer R.A. (1991).

[58] Devilly G.J., Borkovec T.D. (2000). Psychometric properties of the credibility/expectancy questionnaire. J. Behav. Ther. Exp. Psychiatr..

[59] Richards D., Timulak L. (2013). Satisfaction with therapist-delivered vs. self-administered online cognitive behavioural treatments for depression symptoms in college students. Br. J. Guid. Counsell..

[60] Muthén L.K., Muthén B.O. (2015).

[61] Enders C.K. (2010).

[62] Hu L., Bentler P.M. (1999). Cutoff criteria for fit indexes in covariance structure analysis: conventional criteria versus new alternatives. Struct. Equ. Model.: A Multidiscip. J..

[63] Lipsey M.W., Wilson D.B. (2001).

[64] Preacher K.J. (2008).

[65] Muthén L.K., Muthén B.O. (2002). How to use a Monte Carlo study to decide on sample size and determine power. Struct. Equ. Model.: A Multidiscip. J..

[66] Whitehead A.L. (2016). Estimating the sample size for a pilot randomised trial to minimise the overall trial sample size for the external pilot and main trial for a continuous outcome variable. Stat. Methods Med. Res..

[67] Julious S.A. (2005). Sample size of 12 per group rule of thumb for a pilot study. Pharmaceut. Stat..

[68] Moyer A., Finney J. (2004). Brief interventions for alcohol problems: factors that facilitate implementation. Alcohol Res. Health.

[69] Cadigan J.M. (2015). Personalized drinking feedback: a meta-analysis of in-person versus computer-delivered interventions. J. Consult. Clin. Psychol..

[70] Kaner E.F. (2017). Personalised digital interventions for reducing hazardous and harmful alcohol consumption in community-dwelling populations. Cochrane Database Syst. Rev..

[71] Olthuis J.V. (2015). CBT for high anxiety sensitivity: alcohol outcomes. Addict. Behav..

[72] Who (2018).

[73] Kazlauskas E. (2017). Challenges for providing health care in traumatized populations: barriers for PTSD treatments and the need for new developments. Glob. Health Action.

[74] Rodriguez L.M. (2015). Remote versus in-lab computer-delivered personalized normative feedback interventions for college student drinking. J. Consult. Clin. Psychol..

[75] Paulus D.J. (2021). Computer-delivered personalized feedback intervention for hazardous drinkers with elevated anxiety sensitivity: a pilot randomized controlled trial. Behav. Res. Ther..

